# Supplementary material for: Analysis of Circulating Tumour Cells in Early-Stage Uveal Melanoma: Evaluation of Tumour Marker Expression to Increase Capture
Source: Cancers (Basel). 2021 Nov 28;13(23):5990. doi: 10.3390/cancers13235990 (PMC8657240; doi:10.3390/cancers13235990)
Supplement: Supplementary file 1 [file cancers-13-05990-s001.zip › cancers-1475663-supplementary.pdf]

Supplementary Materials

Table of Contents

Supplementary Methods	pg. 2
Supplementary Table S1	pg. 4
Supplementary Figure S1	pg. 5
References	pg. 6

Supplementary Methods

UM Specimens and Tissue Microarray Construction

Formalin-fixed, paraffin-embedded (FFPE) blocks from 10 primary choroidal UM treated by enucleation between 2012-2014 at Royal Perth Hospital, Western Australia, were used to construct the tissue microarray (TMA). A waiver of consent was obtained for all archived tissue blocks under approved Human Research Ethics Committee protocols from Edith Cowan University (No. 12593), Western Australia. The TMA was generated using the TMA Master Tissue Microarrayer (3DHistech). Duplicate (8 patients) and quadruplicate (2 patients) 1 mm cores were taken from areas with high tumour content designated by the pathologist (TV). Non-UM control tissues were obtained from FFPE cutaneous melanoma and normal tonsil, liver, lung, breast, and skin.

Antibody-Bead Coupling

Antibodies (BD Biosciences, **Table S1**) were covalently bound to magnetic beads using a Dynabead Antibody Coupling Kit (Invitrogen) following manufacturer's instructions. 10µg of antibody was used per mg of Dynabeads.

Multiplex Ligation-Dependant Probe Amplification for Detection of Copy Number Variants

To determine chromosomal copy number variations (CNVs), DNA was extracted from FFPE UM specimens by proteinase K digestion and purification through spin columns (Qiagen). For MLPA analysis, 50-120ng DNA was analysed using a SALSA P027-C1 UM probemix kit according to the manufacturer's instructions (MRC Holland, Amsterdam, Netherlands). Samples were separated by capillary electrophoresis on an ABI-3730XL DNA Analyzer (Applied Biosystems) and analysed using Coffalyser software (MRC Holland) to determine copy number changes on chromosomes 1p, 3, 6 and 8.

Circulating Tumour Cell Capture and Quantification in Healthy Controls

Healthy control blood from 10 donors, 6 male and 4 females, with a mean age of 33 (range 23 - 45) were processed using the multimarker approach as per the protocol described in the materials and methods "Circulating tumour cell capture and quantification". No circulating tumour cells, defined as MART1/gp100/S100β positive, CD45/16 negative, Hoechst positive, were detected in these individuals.

Supplementary Table S1: Antibody Information

Antibody	Vendor	Product Code	Use	Dilution
5HT2B	Alomone	ASR-035	IHC, FACS	1:400 (IHC);<br>1:100 (FACS)
ABCB5	Abcam	ab140667	IHC, FACS, CTC	1:100<br>10 µg (beads)
gp100 (BETEB)	NOVUS	NBP2-33172	ICC, FACS, CTC	1:100<br>10 µg (beads)
gp100	Abcam	ab137062	IHC, CTC	1:50
MART1	Abcam	ab51061	IHC, CTC, ICC	1:100 (ICC)<br>1:1000 (IHC)<br>1:50 (CTC)
MCAM	N/A (donated)*	CC9 (clone)	IHC	1:20
MCAM	BD Biosciences	550314	CTC, FACS	1:100<br>10 µg (beads)
MCSP	NOVUS	NB100-2688	IHC, FACS	1:100
MCSP	BD Biosciences	554275	CTC, FACS	1:100<br>10 µg (beads)
Nestin	Abcam	ab18102	IHC, ICC	1:100
RANK	Abcam	ab13918	IHC	1:100
S100β	Abcam	ab4066	IHC, ICC, CTC	1:500
CD45-AF647	BioLegend	304018	CTC	1:20
CD16-AF647	BioLegend	302020	CTC	1:20
Rabbit Isotype	Abcam	ab172730	ICC, FACS	1:100
Mouse Isotype	Abcam	ab18437	ICC, FACS	1:100
Anti-Rabbit AF488	Abcam	ab150065	ICC, FACS	1:500
Anti-Mouse AF488	Abcam	ab150109	ICC, FACS	1:500
Anti-Rabbit AF488	Thermo Fisher Scientific	A-21206	CTC	1:500

Immunohistochemistry (IHC), Immunocytochemistry (ICC), Flow Cytometry (FACS), Circulating Tumour Cell (CTC) isolation (10 µg per mg for coupling) or visualisation.
* CC9 clone was a gift from Dr Danielle Dye, Curtin University [1,2]

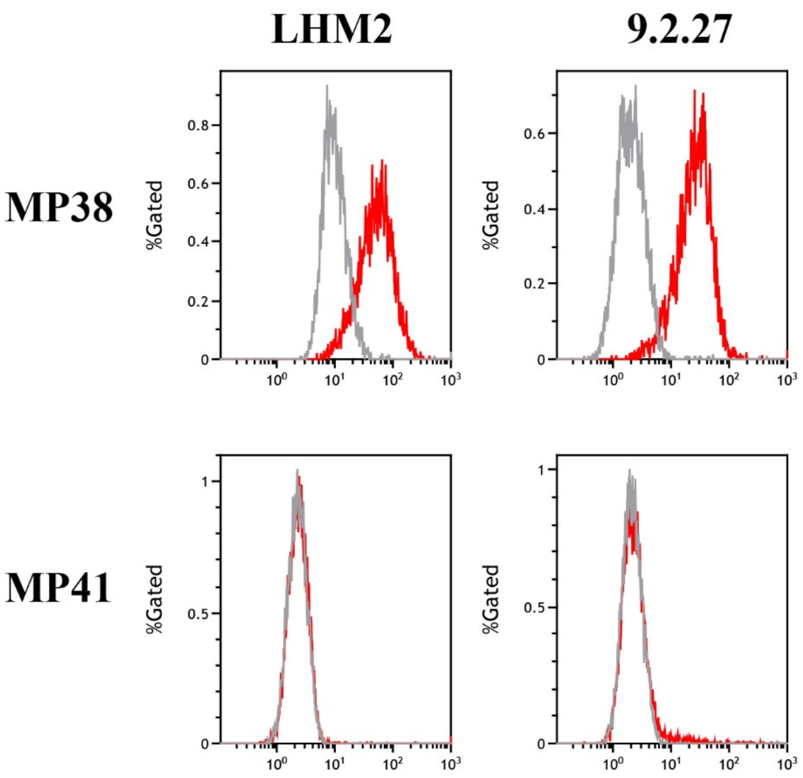

Supplementary Figure S1: Comparison of LHM2 and 9.2.27 MCSP Antibody Clones.

MCSP clones LHM2 and 9.2.27 were used to immunostain MP38 and MP41 cells to ensure both antibodies were capable of detecting MCSP in UM cells. Red profiles indicate MCSP positivity and grey profiles represent negative controls using mouse IgG.

References

1. Dye, D.E.; Karlen, S.; Rohrbach, B.; Staub, O.; Braathen, L.R.; Eidne, K.A.; Coombe, D.R. hShroom1 links a membrane bound protein to the actin cytoskeleton. *Cell Mol Life Sci* **2009**, *66*, 681-696, doi:10.1007/s00018-009-8645-1.
2. Filshie, R.J.; Zannettino, A.C.; Makrynikola, V.; Gronthos, S.; Henniker, A.J.; Bendall, L.J.; Gottlieb, D.J.; Simmons, P.J.; Bradstock, K.F. MUC18, a member of the immunoglobulin superfamily, is expressed on bone marrow fibroblasts and a subset of hematological malignancies. *Leukemia* **1998**, *12*, 414-421.
